# From bridges to cycles in spectroscopic networks

**DOI:** 10.1038/s41598-020-75087-5

**Published:** 2020-11-10

**Authors:** P. Árendás, T. Furtenbacher, A. G. Császár

**Affiliations:** 1 Budapest Business School, Budapest, Hungary; 2 MTA-ELTE Complex Chemical Systems Research Group, Budapest, Hungary; 3 Institute of Chemistry, ELTE Eötvös Loránd University, Budapest, Hungary

**Keywords:** Applied mathematics, Quantum chemistry, Optical spectroscopy

## Abstract

Spectroscopic networks provide a particularly useful representation of observed rovibronic transitions of molecules, as well as of related quantum states, whereby the states form a set of vertices connected by the measured transitions forming a set of edges. Among their several uses, SNs offer a practical framework to assess data in line-by-line spectroscopic databases. They can be utilized to help detect flawed transition entries. Methods which achieve this validation work for transitions taking part in at least one cycle in a measured spectroscopic network but they do not work for bridges. The concept of two-edge-connectivity of graph theory, introduced here to high-resolution spectroscopy, offers an elegant approach that facilitates putting the maximum number of bridges, if not all, into at least one cycle. An algorithmic solution is shown how to augment an existing spectroscopic network with a minimum number of new spectroscopic measurements selected according to well-defined guidelines. In relation to this, two metrics are introduced, ranking measurements based on their utility toward achieving the goal of two-edge-connectivity. Utility of the new concepts are demonstrated on spectroscopic data of $^{14} {\text {NH}}_3$.

## Introduction

Accurate line-by-line (LBL) spectroscopic data are utilized in a wide range of scientific and engineering disciplines with many important applications^1–16^. Accordingly, high-resolution spectroscopic data measured for gas-phase molecules are collected and maintained in large databases, such as the canonical HITRAN spectroscopic information system^16^. Unfortunately, LBL databases may contain rovibronic transitions with flawed wavenumber data or incompatible labels, both of which may lead to several practical issues. For example, the problems indicated may result in inaccurate or incompatible energy values for rovibronic quantum states the LBL data determine. Identification of flawed data entries is an important task during the maintenance and extension of LBL spectroscopic databases.

Spectroscopic networks (SN)^17–21^ offer a novel way to represent the structure of the rovibronic quantum states and the allowed transitions of a molecule. The concept of SNs can be utilized to compare a SN built from data of a LBL database to another one built, for example, from first-principles data available for the same molecule. This comparison offers a powerful approach to gauge the completeness and validity of the data in the database and a framework for using elements of network theory for improving the LBL dataset, in particular to detect its flawed entries.

Let us clarify what is meant here by flawed wavenumber data. Each measured transition in a spectroscopic database has a wavenumber *w*, and a related, preferably two-sigma, uncertainty *u*. (Sometimes, uncertainties are given as uncertainty boundaries, see for example the wavenumber error code labels in HITRAN.) If set properly, together they mean that the “real” wavenumber value should lie, with a probability of 95%, within the $(w-u, w+u)$ interval. Due to human or numerical errors during the buildup of the database, the real wavenumber may lie outside of this interval. While acknowledging that this event has a probability of 5%, we still label this wavenumber datum as *flawed* and suggest that further investigation is needed to verify its correctness.

In a recent paper Tóbiás et al.^21^ have shown how spectroscopic networks can be used to detect flawed wavenumber data in a spectroscopic database. This method, referred to as Cycle Testing in the present paper, works only for transitions which participate in at least one cycle of the SN. Correctness of wavenumber data for transitions outside of cycles cannot be checked this way. The remedy should come in the form of an approach which would put the maximum number of transitions, if not all, into at least one cycle, by adding carefully selected new transitions to the database. At the same time, minimizing the required number of new transitions is extremely important, as this would minimize the associated cost of measuring these transitions. To extend the utility of Cycle Testing, this paper introduces the concept of two-edge-connectivity of graph theory to high-resolution spectroscopy. Two-edge-connectivity offers an elegant approach to improve the basis of Cycle Testing. Since this paper uses various methods and considerations of graph (network) theory, a field which may be somewhat unfamiliar to some of the readers, the authors would like to recommend two outstanding textbooks that establish the required mathematical background^22,23^.

The rest of this paper is organized as follows. “Spectroscopic networks” describes the concept of spectroscopic networks and the notations used, and briefly explains Cycle Testing. It also contains an analysis, from the viewpoint of this paper, of the LBL data of the $^{14} {\text {NH}}_3$ molecule listed in the HITRAN 2016 information system^16^. “Two-edge-connectivity” explains the concept of two-edge-connectivity and its relevance to spectroscopic networks. “Augmenting measured spectroscopic network” formulates the mathematical problem of augmenting the measured spectroscopic network so that it would contain the maximum number of its edges in cycles while adding the minimum number of new transitions to the database. An algorithmic solution of the problem is shown, using a reduction to the (weighted) Tree Augmentation Problem of graph theory^24–29^, in the “Augmenting measured spectroscopic network” section. “Local and global optimality metrics” introduces two metrics to measure the usefulness of the set of new transitions in solving the problem formulated in “Augmenting measured spectroscopic network” section. “Utilization of the concept of two‑edge‑connectivity” illustrates the utility of the concept of two-edge-connectivity on two examples, both involving $^{14} {\text {NH}}_3$. The conclusions reached during this study are summarized in “Conclusions”.

## Spectroscopic networks

### Definitions

The *spectroscopic network* of a molecule is a large graph, whose vertices correspond to rovibronic quantum states and each edge corresponds to a transition between two quantum states allowed by certain so-called selection rules^17^. The energy of the quantum states and the wavenumber and intensity values of the transitions can be taken as weight functions on the vertices and edges, respectively. A distinctive feature of SNs is that wavenumbers on the edges form a potential difference function, using the energy of the vertices as the potential.

**Figure 1:**
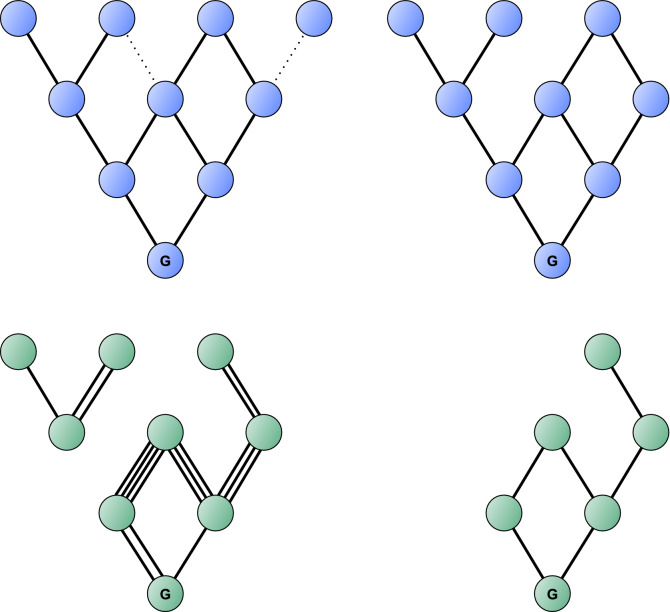
Illustration of the graphs introduced in “Spectroscopic networks” with graphs in the top and bottom rows corresponding to theoretical and measured spectroscopic networks (SN), respectively. Top left panel: graph $SN_{\mathrm{t}}$. Top right panel: graph ${\mathcal {T}}$. Bottom left panel: graph $SN_{\mathrm{m}}$. Bottom right panel: graph ${\mathcal {M}}$. Vertex *G* represents the ground state. Dotted edges of $SN_{\mathrm{t}}$ represent transitions with intensity values below a set $\kappa$ value. These are removed in ${\mathcal {T}}$, as well as the vertices that can only be reached from the ground state through these edges. The parallel edges of $SN_{\mathrm{m}}$ represent the data from multiple sources about the same transition within the experimental database. Observe that in ${\mathcal {M}}$ the parallel edges are replaced with a single edge, and ${\mathcal {M}}$ retains only the connected component of the ground state from $SN_{\mathrm{m}}$. It can also be seen that ${\mathcal {M}}$ is a subgraph of ${\mathcal {T}}$.

The vertex-edge structure of spectroscopic networks is determined by appropriate selection rules, which may be different for different experimental techniques. As to the energies and the weights of the transitions, they are known only approximately. There are two principal ways to obtain wavenumber (and intensity) data: a theoretical (preferably first-principles^30^) and an experimental (preferably ultra-high precision^31^) one. Both approaches have their own advantages and disadvantages. The first-principles approach solves the nuclear Schrödinger equation of quantum chemistry numerically^30^ and computes approximate wavenumbers (and intensities) for perhaps all feasible transitions, though with relatively sizeable error margins, often several orders of magnitude larger than the uncertainties of modern experiments^31^. In the experimental method accurate wavenumber (and intensity) data are obtained from measured spectra, but only about a (small) subset of all transitions. Experimental data about a molecule are usually collected in (large) spectroscopic databases.

A spectroscopic network that is built from first-principles data is called a *theoretical spectroscopic network*. If the transitions forming the SN come from experiment, it is called a *measured spectroscopic network*. Modern LBL databases may also contain transitions that do not come from experiment but have theoretical/computational origin. For example, the HITRAN database contains data from effective Hamiltonian fits. This issue is rectified by relaxing the definition of the measured spectroscopic network as follows. As the goal of spectroscopic databases, like HITRAN, is to provide data sets with accuracy that is comparable to genuine experimental data, the graphs built using them will still be considered measured. Thus, “measured SNs” may contain accurate transitions of theoretical origin, alongside the experimental data. Throughout this paper, the intensities of the rovibronic transitions of ammonia ($^{14} {\text {NH}}_3$), our test molecule, refer to room temperature (296 K).

Let us denote the theoretical spectroscopic network built from vertices *V* and edges *E* by $SN_{\mathrm{t}}=(V_{\mathrm{t}},E_{\mathrm{t}})$ and the measured spectroscopic network by $SN_{\mathrm{m}}=(V_{\mathrm{m}},E_{\mathrm{m}})$. There is no need to denote weight functions on the two graphs. Observe that $V_{\mathrm{m}} \subseteq V_{\mathrm{t}}$, and $E_{\mathrm{m}}$ may contain parallel edges.

For practical reasons, graphs ${\mathcal {T}}$ and ${\mathcal {M}}$ will be defined and used instead of $SN_{\mathrm{t}}$ and $SN_{\mathrm{m}}$, respectively (see Fig. 1). Briefly, graph ${\mathcal {T}}$ will only contain transitions above an intensity threshold dictated by feasible measurements, while graph ${\mathcal {M}}$ will be a connected graph without any parallel edges.

Let us define the graph ${\mathcal {T}}=(V_{\mathcal {T}},E_{\mathcal {T}})$ as follows. The edge set $E_{\mathcal {T}}$ is the set of all transitions in $E_{\mathrm{t}}$ that have at least an intensity value of $\kappa$. The $\kappa$ parameter corresponds to the smallest intensity value by which the transition can be detected in the measured spectrum of the molecule. For example, $\kappa =10^{-30} \, \hbox {cm}\, {\hbox {molecule}}^{-1}$ is a typical lower limit of cavity-ringdown spectroscopic measurements, some of the most sensitive techniques of modern-day high-resolution spectroscopy^32^. The vertex set $V_{\mathcal {T}}$ is the subset of $V_{\mathrm{t}}$ to which at least one transition belongs from $E_{\mathcal {T}}$.

Let us define the graph ${\mathcal {M}}=(V_{\mathcal {M}},E_{\mathcal {M}})$ as follows. Let $V_{\mathcal {M}}$ be the subset of vertices in $V_{\mathrm{m}}$ which are in the same connected component as the ground state in $SN_{\mathrm{m}}$. Then, for all $u,v \in V_{\mathcal {M}}$, there is a single (*u*, *v*) edge in $E_{\mathcal {M}}$ if there is at least one (*u*, *v*) edge in $E_{\mathrm{m}}$. Observe that ${\mathcal {M}}$ is a subgraph of ${\mathcal {T}}$, or in other words, $V_{\mathcal {M}} \subseteq V_{\mathcal {T}}$ and $E_{\mathcal {M}} \subseteq E_{\mathcal {T}}$.

**Figure 2:**
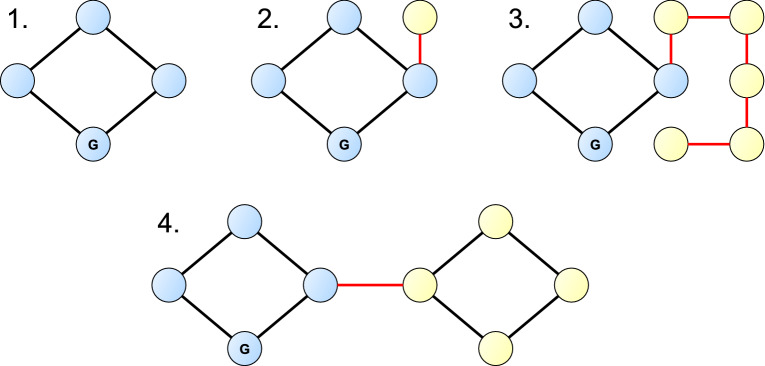
A graph without bridges (no. 1) and three graphs that do contain bridges (no. 2–4). Graph no. 2 has one bridge, ending in a one-degree vertex (a vertex that has only one edge, a leaf). Graph no. 3 has 5 bridges together forming a branch, one of which ends in a 1$^{\circ }$ vertex. Graph no. 4 has one bridge, that connects two subgraphs without bridges. In all graphs blue vertices are reachable from vertex *G*, representing the ground state, through a path that does not contain bridges; yellow vertices can only be reached from vertex *G* through at least one bridge.

### Cycle testing

As shown by Tóbiás et al.^21^, the cycles of measured SNs can be utilized to detect flawed wavenumber data entries in experimental line-by-line spectroscopic databases. A cycle is a subset of the edges that form a path in the graph that starts and ends in the same vertex. Using Cycle Testing, one can detect flawed transitions in cycles in measured SNs. Let us call the edges that are not in at least one cycle in the graph *bridges*. Thus, Cycle Testing can be done for all edges that are not bridges. Figure 2 shows an example of a graph without any bridges, and three examples of graphs with various types of bridges. Cycle Testing is based on the fact that wavenumbers, as a weight function, form a potential difference function on the edges. Briefly, Cycle Testing checks this attribute for the cycles of a given measured SN as follows.

Let us take a cycle from the measured SN that has no parallel edges. Direct the edges from the lower energy vertex to the upper energy vertex, perhaps based on the corresponding theoretical SN. The signed sum of wavenumbers along a cycle means the sum of wavenumbers, with each edge that is traversed from its head to its tail counting as a negative number. If one can select one wavenumber for each edge from its wavenumber interval such that the signed sum along the cycle is zero, within the tolerance of the associated uncertainties, then the cycle is *consistent*. Else, the cycle is *inconsistent*, and it contains at least one edge with flawed wavenumber data. Testing additional cycles could help narrowing down the possible edge set with flawed data. Consistency and inconsistency can also be defined for the full SN.

**Figure 3:**
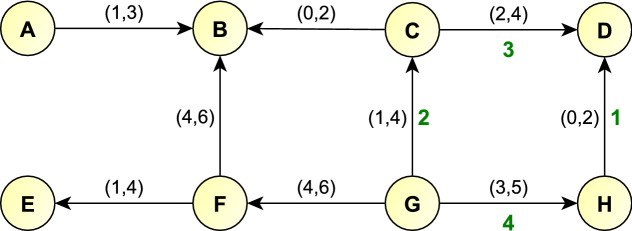
Illustration of bridges and Cycle Testing on a small graph. Each edge, except edges AB and EF, is present in at least one cycle. Thus, edges AB and EF are the bridges of the graph. Edge labels: wavenumber intervals (black), a possible zero-signed-sum wavenumber selection for the *GCDH* cycle (green). The *GCDH* cycle is consistent, the *GFBC* cycle is inconsistent.

Let us demonstrate how Cycle Testing works on the graph of Fig. 3. There are wavenumber intervals given on the edges of the graph, which correspond to the $(w-u,w+u)$ intervals introduced in “ Introduction”. For example, the wavenumber interval (1, 3) corresponds to $w=2$ and $u=1$. The small graph of Fig. 3 contains three cycles. According to the wavenumber intervals of the edges, there exists a zero-signed-sum wavenumber selection on the *GCDH* cycle, for example, $2+3-1-4=0$ (for the sake of simplicity, uncertainties much larger than real-life ones are assumed). However, for the *GFBC* cycle no such wavenumber set exists; for example, the minimum wavenumber sum on the edges *GF* and *FB* is $4+4=8$, while the maximum wavenumber sum on the edges *GC* and *CB* is $4+2=6$. Therefore, the *GCDH* cycle is consistent, while the *GFBC* cycle is inconsistent.

In practice, a considerable drawback of Cycle Testing is the time complexity of cycle-finding algorithms. Finding all cycles of a graph, for example, is a NP-complete problem, meaning that no “quick” solution exists. Finding cycles of a fixed length of *k* is a different problem, but for $k \ge 6$ the algorithms become rather slow, given the large amount of vertices and edges in SNs. Thus, Cycle Testing is advocated to be used with cycles of length 4, consideration of cycles of length 6 and 8 is only advocated for small subgraphs of SNs (note, in this respect, that all SNs are bipartite graphs).

### Experimental data

Bridges have a direct impact on the overall consistency and utility of an experimental database. Let us investigate this impact employing data from the HITRAN 2016 dataset^16^ on the $^{14} {\text {NH}}_3$ isotopologue of ammonia. There are two principal components (PC)^20^ of this spectroscopic network, corresponding to two nuclear-spin isomers: one contains the transitions among the *ortho*-$^{14} {\text {NH}}_3$ states, while the other among the *para*-$^{14} {\text {NH}}_3$ states. Let us consider the two graphs that are obtained from the experimental data following the graph construction of graph ${\mathcal {M}}$ described in “Definitions”. Only the transitions between quantum states with the complete label containing 13 descriptors^33–36^ were used during this construction. Table 1 displays the numerical data upon which the upcoming analysis is built.

**Table 1:** Selected spectroscopic data characterizing the $^{14} {\text {NH}}_3$ molecule in the HITRAN 2016 information system^16^.

	*ortho*-$^{14} {\text {NH}}_3$	*para*-$^{14} {\text {NH}}_3$
No. of edges (unique transitions)	13105	28704
No. of bridges	619 (4.7%)	879 (3%)
No. of vertices (quantum states)	2189	3978
No. of vertices reachable from the ground state only through at least one bridge	619 (28%)	879 (22%)
No. of one-degree vertices	616	878

First, let us observe that in our example the ratio of the number of bridges to the total number of edges is low: $4.7\%$ in the *ortho* and $3\%$ in the *para* cases. Thus, the vast majority of transitions can be verified by Cycle Testing for both principal components of the measured SN.

Next, let us determine the number of vertices (quantum states) that can only be reached from the ground state on a path that contains at least one bridge. The computed energy of these quantum states depends on at least one wavenumber that is unverifiable by Cycle Testing. In the *ortho* case, this is $28\%$ of all quantum states, while in the *para* case $22\%$ of all quantum states belong to this category. In summary, the ratio of quantum states whose energy value cannot be verified by Cycle Testing is high, and this is caused by a relatively small subset of edges. Note that this observation can also be made on graph no. 4 of Fig. 2. This graph contains only one bridge, which is only about $11\%$ of all edges in the graph. However, if vertex *G* represents the ground state, then $50\%$ of the vertices can only be reached from the ground state using this bridge.

Note also that in our example the number of one-degree vertices is almost equal to the number of bridges for both *ortho*- and *para*-$^{14} {\text {NH}}_3$. This means that almost all bridges have an endpoint with a degree of 1. Two bridges with this property can be seen in Fig. 2: one in graph no. 2 and the other in graph no. 3. If a bridge has an endpoint with a degree of 1 (a so-called leaf), then only this one-degree vertex can be reached through this bridge; thus, this bridge does not affect the consistency of the rest of the network. This distinction of bridges is important from the viewpoint that a bridge could easily affect the consistency of large subgraphs. For an example, see graph no. 4 of Fig. 2, where one bridge affects half of the vertices.

Now, adding new transitions to the database (*via* new spectroscopic measurements) could transform bridges into members of cycles. It can be easily determined about a new transition whether it puts at least one bridge into a cycle. However, in general this is not a trivial problem. Furthermore, some of the new transitions can be more useful than others. As obtaining new transitions often involves substantial cost, to select a set of new transitions to add to the database becomes an optimization problem. The best set of new transitions improves the consistency of the database with a reasonable incurring cost. In the case of the analysis corresponding to Table 1, given the low ratio of bridges among edges, it is expected that the consistency of the SN can be improved by adding just a small number of new transitions to it.

## Two-edge-connectivity

In graph theory there are multiple equivalent conditions to the property that each edge is in at least one cycle in a connected graph^22,37,38^. The condition used in this paper is that graphs without bridges are two-edge-connected. This is the key graph property utilized and explored in this paper.

A graph is *k*-edge-connected if there are at least *k* edge-disjoint paths (i.e., paths without common edges) between each vertex pair in the graph. Thus, if there are at least two edge-disjoint paths between each vertex pair in the graph, then each edge of the graph participates in at least one cycle. Figure 2 shows a graph (graph no. 1) that is two-edge-connected, and three other graphs that are not two-edge-connected.

It is easy to see that if a graph is two-edge-connected, then for any edge $e_{ij}$, connecting vertices *i* and *j*, there are at least two edge-disjoint paths in the graph between *i* and *j*. One of these paths is the $e_{ij}$ edge itself, and there is at least one additional path, $P_{ij}$, that does not contain $e_{ij}$. Putting together $e_{ij}$ and $P_{ij}$ we obtain a cycle that includes $e_{ij}$.

If the graph ${\mathcal {M}}$ is two-edge-connected, then it does not contain any bridges. Thus, the energy of all quantum states in the component containing the ground state, whose energy can conveniently be set to zero, are verifiable by Cycle Testing. This is the ideal scenario. From now on, let us assume that ${\mathcal {M}}$ is not two-edge-connected.

While ${\mathcal {M}}$ itself is not a two-edge-connected graph, it can have one or more two-edge-connected subgraphs. For example, the graph shown in Fig. 3 is not two-edge-connected, but it has a two-edge-connected subgraph, which is the subgraph formed by the vertex set $\{B, C, D, F, G, H\}$ and the edges that span between these vertices. A similar property can be observed on graph no. 4. of Fig. 2, where a bridge connects two two-edge-connected graphs.

Each edge in a two-edge-connected subgraph is already included in at least one cycle; in other words, none of the edges of two-edge-connected subgraphs are bridges. This also means that the energy of quantum states in the same two-edge-connected component as the ground state can be verified by Cycle Testing. If in the graph of Fig. 3 vertex G represents the ground state, then the energies of the quantum states $\{B, C, D, F, G, H\}$ can be verified by Cycle Testing. However, the energy of quantum states *A* and *E* cannot be verified in this way: transitions *AB* and *EF* may contain flawed wavenumber data.

Identification of the maximal two-edge-connected subgraphs can be done, for example, by using an efficient algorithm of Tarjan^39^. In a connected graph one can also use Dinic’s algorithm^40^ to count the number of edge-disjoint paths from the ground state to all other quantum states.

## Augmenting measured spectroscopic networks

There is a natural min-max problem related to the process of adding new edges to an existing SN: maximize the number of bridges converted into cycles while minimizing the number of required new transitions. Optimally, all the bridges of the original SN are converted into cycles. However, difficulties could arise, for example, if no new measurable transition could be found for a bridge that would convert it into a member of a cycle. The minimization corresponds to the associated real-life cost of obtaining new transitions. Moreover, the minimization requirement also represents that in real life there may be other goals to consider than forming cycles when suggesting new transitions for measurement.

**Figure 4:**
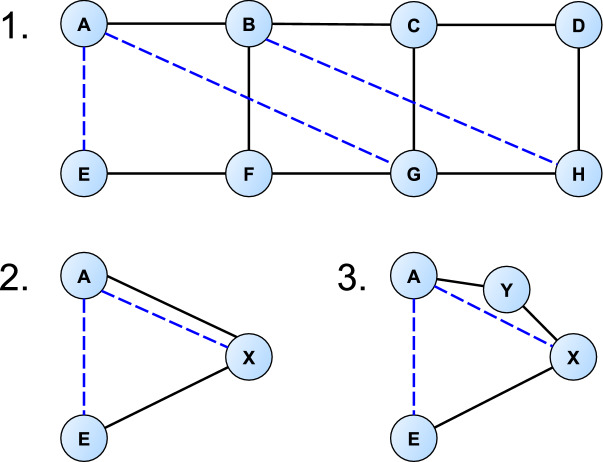
Illustration of the construction steps of graph ${\mathcal {M}}^*$. Graph no. 1 without the blue lines is graph ${\mathcal {M}}$. Blue lines represent the elements of the edge set ${\mathcal {E}}$. Graphs no. 2 and no. 3 represent two important construction steps.

The following graph-construction example let us focus nicely on the bridges of graph ${\mathcal {M}}$. The step-by-step graphical representation of the graph construction is shown in Fig. 4, whereby the graph corresponds to the 8-vertices graph of Fig. 3, with three possible new transitions added, one between *A* and *E*, one between *A* and *G*, and one between *B* and *H*.

Let us denote the vertex sets of the maximal two-edge-connected subgraphs in ${\mathcal {M}}$ by $C_1, \dots , C_m$. Let us denote by ${\mathcal {M}}^*=(V_{\mathcal {M}}^*,E_{\mathcal {M}}^*)$ the graph obtained from ${\mathcal {M}}$ after contracting the spanning subgraphs of vertex sets $C_1, \dots , C_m$ to single vertices $c_1, \dots , c_m$. Clearly, ${\mathcal {M}}^*$ is a tree.

Graph no. 1 of Fig. 4 without the blue edges has one maximal two-edge-connected subgraph, defined by the vertex set $\{B, C, D, F, G, H\}$ and the edges that span between these vertices. Graph no. 2 of Fig. 4 shows what we obtain after we contract this maximal two-edge-connected subgraph to a single vertex *X*. Observe that the possible new edge between vertices *B* and *H* has vanished: it is inside a two-edge-connected component; thus, its addition would not put any bridge into a new cycle.

Let us define the edge set ${\mathcal {E}}=\{(u,v): u \in C_i, v \in C_j, i \ne j, (u,v) \in E_{\mathcal {T}} \setminus E_{\mathcal {M}}\}$. This is the set representing all possible new transitions that could be added to the experimental database, which convert at least one bridge into a new cycle, and which span between two quantum states that are already in the experimental spectroscopic database.

In graph no. 2 of Fig. 4 it can be seen that ${\mathcal {E}}=\{ (A,E), (A,X)\}$, and between vertices *A* and *X* we have parallel edges: one because there is a transition in the experimental database between the two corresponding vertex sets, and the other because there is a feasible new experimental transition between the two corresponding vertex sets. In order not to have to deal with parallel edges, let us modify the edges of ${\mathcal {M}}^*$ by splitting each of its edges where a parallel new edge from ${\mathcal {E}}$ exists by adding a midpoint vertex: if we had an edge betwen $v_1$ and $v_2$, in the new graph we will have an edge between $v_1$ and $v'$, and one between $v'$ and $v_2$, where $v'$ is a new midpoint vertex inserted. Graph no. 3 of Fig. 4 displays the step of splitting the edge between vertices *A* and *X* by the new midpont vertex *Y*.

Now, if the addition of all edges in ${\mathcal {E}}$ to the graph ${\mathcal {M}}^*$ (more precisely, forming the graph $(V_{\mathcal {M}}^*,E_{\mathcal {M}}^* \cup {\mathcal {E}})$) results in a two-edge-connected graph, then ${\mathcal {M}}$ can be augmented with new edges to remove all of its bridges. However, if $(V_{\mathcal {M}}^*,E_{\mathcal {M}}^* \cup {\mathcal {E}})$ is not two-edge-connected, then ${\mathcal {M}}$ cannot be augmented to a two-edge-connected graph this way. To address this issue, one could first determine the two-edge-connected subgraphs of $(V_{\mathcal {M}}^*,E_{\mathcal {M}}^* \cup {\mathcal {E}})$ and do the augmentation of the corresponding subgraphs separately. This way, although not all, but at least a subset of the bridges in ${\mathcal {M}}$ could be put into cycles. From this point on, $(V_{\mathcal {M}}^*,E_{\mathcal {M}}^* \cup {\mathcal {E}})$ is assumed to be a two-edge-connected graph.

Adding ${\mathcal {E}}$ to $E_{\mathcal {M}}$ would trivially make ${\mathcal {M}}$ a two-edge-connected graph. However, an important goal is to select a subgraph of ${\mathcal {E}}$ of minimum size, whose addition to the edge set $E_{\mathcal {M}}$ would make ${\mathcal {M}}$ a two-edge-connected graph. The right-hand graph of the bottom row of Fig. 4 without the blue edges can be augmented to a two-edge-connected graph by adding only the edge between vertices *A* and *E*, adding the other blue edge is not necessary. A feasible solution to this problem is obtained *via* a reduction to the *Tree Augmentation Problem* (TAP) of graph theory^24–29^.

In TAP, the input consists of a tree $T=(V,F)$ and an edge set $E \subseteq V \times V$, where $E \cap F = \emptyset$. The goal is to find a subset $E' \subseteq E$ with the minimum number of edges such that $T'=(V,F \cup E')$ is two-edge-connected. For the reduction, let us set $T={\mathcal {M}}^*$ and $E={\mathcal {E}}$. Solving this problem is equivalent to the problem of selecting a minimum number of new edges ${\mathcal {E}}' \subseteq {\mathcal {E}}$ to put all bridges of the original graph into at least one cycle.

A variant of TAP is the *Weighted Tree Augmentation Problem* (WTAP), where there is a weight function on the edges in *E*. Here, the goal is to find a subset $E' \subseteq E$ with minimum total weight, such that $T'=(V,F \cup E')$ is two-edge-connected. Should a weight function on the expanding edge set ${\mathcal {E}}$ be useful, the Weighted Tree Augmentation Problem offers a convenient approach. For example, edge weights could express the preference that transitions with higher intensity values are generally easier to identify in spectroscopic measurements.

Both TAP and WTAP are known to be NP-hard, but there are various approximation algorithms available. An approximation algorithm in our case means that if *T* could be augmented to a two-edge-connected graph using *m* edges, then, by using an $\alpha$-approximation algorithm, we would obtain at most $\alpha m$ edges that would augment *T* to a two-edge-connected graph.

During the selection of the algorithm to solve TAP (or WTAP), one should consider the properties of the graphs $T={\mathcal {M}}^*$ and $E={\mathcal {E}}$. For arbitrary graphs, TAP can be solved with an approximation ratio of 1.5^24^, and 1.5 is also a lower bound for the LP-relaxation of the problem^25^. If *T* is to be augmented only by edges that connect leaves, better approximations are possible^26^. For WTAP and arbitrary graphs, the best known approximation ratio is 2^27^. Additionally, for WTAP there is a $(1+ \ln 2)$-approximation algorithm for trees with constant radius^28^, and a ${\sim } 1.964\,17$-approximation algorithm if the costs have an upper bound^29^.

It should be noted that both the graph contraction steps and the approximation algorithms of TAP referenced above can be done in polynomial time complexity. We advocate the use of a linear programming model in practice to find an approximate solution of TAP.

## Local and global optimality metrics

A difficulty in the practical solution of making a measured network two-edge-connected arises from the technical constraints of spectroscopic measurements. Rather than measuring the whole spectrum, and thus obtaining information about all transitions of the molecule, spectroscopic measurements only capture data from parts of the spectrum. Resolution and detectability issues aside, a spectrum fragment contains all transitions that have a wavenumber value in a measurement-specific interval. For example, if a measurement captures the spectrum fragment between wavenumbers $w_1$ and $w_2$, a transition with a wavenumber value *w* is captured only if $w \in [w_1,w_2]$. The problem is that ${\mathcal {E}}'$ may contain edges that lie outside of the wavenumber interval of a given measurement.

Let us insert here a short remark concerning the wavenumber intervals of measurements. Previously it was discussed how *ab initio* data can be filtered using an intensity cut-off parameter. Similarly, a wavenumber cut-off can also be employed in the *ab initio* data, resulting in computed transitions that are estimated to lie in the wavenumber interval of the measurement.

It should be added that even if a transition belongs to the wavenumber range of a feasible measurement, there are certain factors that could still prevent the identification of the transition in the spectrum fragment, and thus to obtain its wavenumber value. Most notably, transitions with low intensity values, especially if they overlap with much higher intensity lines, can not be detected in a reliable manner. The problem of low intensities of well separated lines is handled by the parameter $\kappa$ during construction of graph ${\mathcal {T}}$ (see  “Spectroscopic networks”), but other possible issues, like overlapping transitions, are out of the scope of this paper.

If various measurements with different wavenumber intervals could be made about the complete spectrum of a molecule, then the question which are the most useful measurements to augment ${\mathcal {M}}$ to a two-edge-connected graph becomes particularly important. An alternative question is whether the available measurements could be arranged into an ordered list of usefulness in making the measured SN two-edge-connected.

To address these issues, let us introduce two metrics to express the usefulness of a given measurement *M*. Let $f_{\mathrm{g}}(M)$ denote the *global optimality metric* of measurement *M*, and let $f_{\ell }(M)$ denote the *local optimality metric* of measurement *M*. During comparison of two or more measurements based on one of the two metrics, higher values will indicate more useful measurements.

Let us denote the wavenumber interval of the measurement *M* by *W*(*M*) and the wavenumber value of the edge (*u*, *v*) by *w*(*u*, *v*). The metrics $f_{\mathrm{g}}$ and $f_{\ell }$ are defined as follows.

1. Global optimality metric: Let $X=\{(u,v) \in {\mathcal {E}}', w(u,v) \in W(M)\}$. Then, let $f_{\mathrm{g}}(M)=|X|$.
2. Local optimality metric: Let ${\mathcal {E}}\vert _{M}=\{(u,v): (u,v) \in {\mathcal {E}}, w(u,v) \in W(M)\}$. Let us assume that $(V_{\mathcal {M}}^*,E_{\mathcal {M}}^* \cup {\mathcal {E}}\vert _{M})$ is two-edge-connected. If it is not, determine its two-edge-connected subgraphs, then solve the problem for these subgraphs separately. Then, solve the Tree Augmentation Problem by setting $T={\mathcal {M}}^*$ and $E={\mathcal {E}}\vert _{M}$, that results in ${\mathcal {E}}\vert _{M}'$. Let $G=(V_{\mathcal {M}},E_{\mathcal {M}} \cup {\mathcal {E}}\vert _{M}')$. Let us denote the number of edges that are in at least one cycle in the graph *G* by *c*(*G*), and let us denote the number of edges in ${\mathcal {E}}\vert _{M}'$ by *e*. Then, let $f_{\ell }(M)=c(G)-e-c({\mathcal {M}})$.

**Figure 5:**
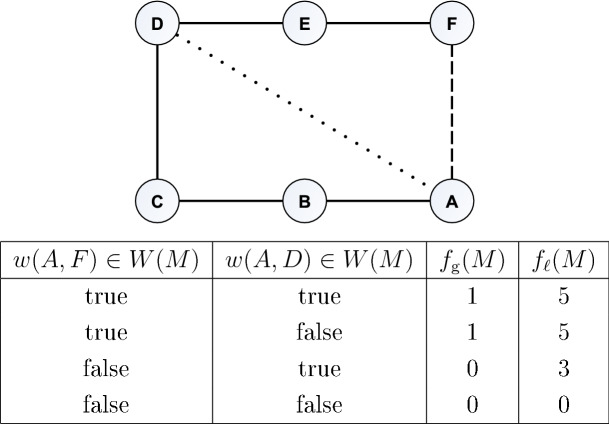
Calculation of the global and local optimality metrics $f_{\mathrm{g}}$ and $f_{\ell }$, respectively, of a small example graph, where ${\mathcal {E}}'=\{(A,F)\}$.

Briefly, the global optimality metric $f_{\mathrm{g}}$ counts the number of new edges provided by the measurement that belong to ${\mathcal {E}}$ (the minimum set of edges that make ${\mathcal {M}}$ a two-edge-connected graph). Meanwhile, the local optimality metric $f_{\ell }$ counts the number of edges not in cycles in ${\mathcal {M}}$ that could be put into at least one cycle by the new edges provided by the measurement. Figure 5 shows the $f_{\mathrm{g}}$ and $f_{\ell }$ values of a measurement *M* on the example of a small graph.

## Utilization of the concept of two-edge-connectivity

In this section we demonstrate the utility of the concept of two-edge-connectivity on high-resolution spectroscopy *via* two examples. Both concern the $^{14} {\text {NH}}_3$ molecule, but they differ in their goals.

The first example demonstrates the general principles and considerations of our method. In order to do this, both a SN and a set of extra edges is created synthetically from the HITRAN data on $^{14} {\text {NH}}_3$^16^. The augmentation problem obtained this way illustrates nicely how the selection of new edges works.

The second example is a practical application of our method, which is used to suggest new edges to be added to the MARVEL^33^ data of $^{14} {\text {NH}}_3$, to improve the calculated energy of a considerable number of quantum states.

### A synthetic example

For our first example, let us construct a measured spectroscopic network ${\mathcal {M}}_1$ and a corresponding set of extra edges ${\mathcal {E}}_1$, and let us see how the addition of edges from ${\mathcal {E}}_1$ to ${\mathcal {M}}_1$ places some of the bridges of ${\mathcal {M}}_1$ into cycles. The underlying data for both ${\mathcal {M}}_1$ and ${\mathcal {E}}_1$ come from the transition list of the $^{14} {\text {NH}}_3$ molecule in the HITRAN 2016 information system^16^. This source was already mentioned and discussed in “Experimental data” and Table 1.

First, let us consider from Table 1 the SN component corresponding to *ortho*-$^{14} {\text {NH}}_3$. Let us denote this graph by ${\mathcal {M}}_\text {ortho}$. The graph ${\mathcal {M}}_\text {ortho}$ contains 13 105 edges. Next, let $E_{\mathrm{S}}$ denote the subset of edges of the graph ${\mathcal {M}}_\text {ortho}$ that span between either two quantum states with vibrational symmetry labels ${\hbox {E}}'$ (597 edges) or a quantum state with a vibrational symmetry label of ${\hbox {E}}'$ and another with a vibrational symmetry label of ${\hbox {A}}_{1}'$ (1743 edges). Let us denote the endpoints of the edges in $E_{\mathrm{S}}$ by $V_{\mathrm{S}}$. Then, let us define the graph ${\mathcal {M}}_1=(V_{\mathrm{S}},E_{\mathrm{S}})$.

The graph ${\mathcal {M}}_1$ contains 821 vertices and 2340 edges, from which 300 edges are bridges. In fact, in ${\mathcal {M}}_1$ there is a central two-edge-connected component and 300 one-degree vertices. The high ratio of bridges to all edges is expected, as ${\mathcal {M}}_\text {ortho}$ also contains a lot of bridges.

Let us define a set of extra edges that can place some of the bridges of ${\mathcal {M}}_1$ into cycles. For this, let ${\mathcal {E}}_1$ denote the subset of edges of the graph ${\mathcal {M}}_\text {ortho}^{^{14}\text {NH}_3}$ that span between two quantum states with vibrational symmetry labels ${\hbox {A}}_{1}'$ (1374 edges).

It should be noted that there are edges in ${\mathcal {E}}_1$ that have their endpoints outside of ${\mathcal {M}}_1$. This is because the graph ${\mathcal {M}}_1$ only contains the quantum states with vibrational symmetry label ${\hbox {A}}_{1}'$ that have direct connections to quantum states with vibrational symmetry label ${\hbox {E}}'$; however, there are ${\hbox {A}}_{1}'$–${\hbox {A}}_{1}'$ transitions between quantum states that are not directly connected to at least one ${\hbox {E}}'$ state. These edges of ${\mathcal {E}}_1$ cannot be used to put bridges of ${\mathcal {M}}_1$ into cycles without adding new vertices to ${\mathcal {M}}_1$; thus, they are discarded. After this, ${\mathcal {E}}_1$ contains 53 unique transitions.

Now, let us contract the central two-edge-connected component of ${\mathcal {M}}_1$ to a single vertex, as described in “Augmenting measured spectroscopic networks”, and let us denote the graph obtained this way by ${\mathcal {M}}^*_1$. The graph ${\mathcal {M}}^*_1$ contains 301 edges, as expected, since the graph ${\mathcal {M}}_1$ contains 300 bridges. The shape of ${\mathcal {M}}^*_1$ is a star, it has one central vertex and 300 one-degree vertices.

**Figure 6:**
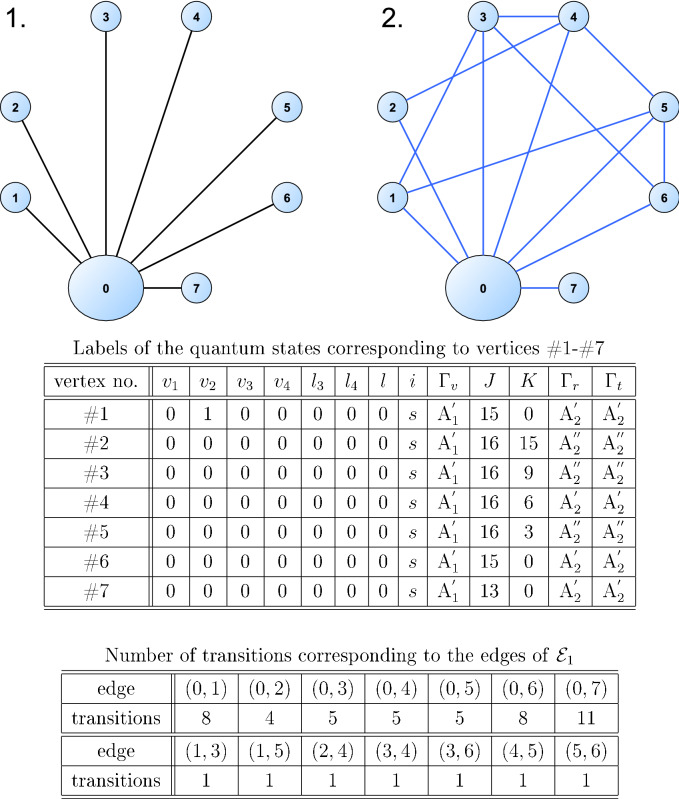
Visual representation and data about the graph construction of “Utilization of the concept of two-edge-connectivity”. Left graph (1.): a subgraph of ${\mathcal {M}}^*_1$. Right graph (2.): the position of the edges of ${\mathcal {E}}_1$ in the graph ${\mathcal {M}}^*_1$ after contraction. See Ref.^34^ for the meaning of the quantum descriptors.

After this contraction, the 53 edges of ${\mathcal {E}}_1$ now correspond to edges between 14 vertex pairs of ${\mathcal {M}}^*_1$, spanning among eight vertices of ${\mathcal {M}}^*_1$. One of these eight vertices originates from the contracted subgraph; the other seven vertices are unique quantum states.

A visual representation of the graph construction is shown in Fig. 6. Both graphs of Fig. 6 show the central vertex of ${\mathcal {M}}^*_1$, labeled as vertex #0, and the other seven vertices of ${\mathcal {M}}^*_1$, which are together the endpoints of the edges of ${\mathcal {E}}_1$. Vertices of ${\mathcal {M}}^*_1$ which are not endpoints of the edges of ${\mathcal {E}}_1$ are not shown. The edges of the left graph (in black) show the edges of ${\mathcal {M}}^*_1$; the edges of the right graph (in blue) show the edges of ${\mathcal {E}}_1$ after contraction. The spectroscopic designation of the quantum states corresponding to vertices #1–#7 are displayed in the top table.

Figure 6 displays the edges of ${\mathcal {E}}_1$ after contraction (note that each of these edges may correspond to multiple transitions). The bottom table of Fig. 6 shows the number of such ${\hbox {A}}_{1}'$–${\hbox {A}}_{1}'$ transitions that span between vertex pairs of ${\mathcal {M}}^*_1$. According to this table, for example, the edge between vertices #0 and #1 corresponds to a set of eight unique transitions.

The positions of the edges of ${\mathcal {E}}_1$ after the contraction in Fig. 6 indicate that by adding all 53 edges of ${\mathcal {E}}_1$ to the graph ${\mathcal {M}}_1$ would put seven bridges of ${\mathcal {M}}_1$ into cycles, lowering the total number of bridges to 293. However, the number of the extra edges required to put these seven bridges into cycles can be lowered in two steps.

**Table 2:** Quantum states belonging to vertex #0 of the graph ${\mathcal {M}}^*_1$ of Fig. 6, that are connected to the quantum state corresponding to vertex #7 of the graph ${\mathcal {M}}^*_1$.

$v_1$	$v_2$	$v_3$	$v_4$	$l_3$	$l_4$	*l*	*i*	$\Gamma _v$	*J*	*K*	$\Gamma _r$	$\Gamma _t$
0	0	0	0	0	0	0	*s*	${\hbox {A}}_{1}'$	14	9	${\hbox {A}}_{2}''$	${\hbox {A}}_{2}''$
0	0	0	0	0	0	0	*s*	${\hbox {A}}_{1}'$	13	3	${\hbox {A}}_{2}''$	${\hbox {A}}_{2}''$
0	0	0	0	0	0	0	*s*	${\hbox {A}}_{1}'$	14	3	${\hbox {A}}_{2}''$	${\hbox {A}}_{2}''$
0	0	0	0	0	0	0	*s*	${\hbox {A}}_{1}'$	12	3	${\hbox {A}}_{2}''$	${\hbox {A}}_{2}''$
0	0	0	0	0	0	0	*s*	${\hbox {A}}_{1}'$	13	9	${\hbox {A}}_{2}''$	${\hbox {A}}_{2}''$
0	1	0	0	0	0	0	*s*	${\hbox {A}}_{1}'$	12	9	${\hbox {A}}_{2}''$	${\hbox {A}}_{2}''$
0	0	0	0	0	0	0	*s*	${\hbox {A}}_{1}'$	12	9	${\hbox {A}}_{2}''$	${\hbox {A}}_{2}''$
0	1	0	0	0	0	0	*s*	${\hbox {A}}_{1}'$	13	9	${\hbox {A}}_{2}''$	${\hbox {A}}_{2}''$
0	1	0	0	0	0	0	*s*	${\hbox {A}}_{1}'$	12	3	${\hbox {A}}_{2}''$	${\hbox {A}}_{2}''$
0	1	0	0	0	0	0	*s*	${\hbox {A}}_{1}'$	13	3	${\hbox {A}}_{2}''$	${\hbox {A}}_{2}''$
0	1	0	0	0	0	0	*s*	${\hbox {A}}_{1}'$	14	3	${\hbox {A}}_{2}''$	${\hbox {A}}_{2}''$

See Ref.^33^ for the meaning of the column headings.

First, as the edges of ${\mathcal {E}}_1$ span between 14 vertex pairs of ${\mathcal {M}}^*_1$, adding one edge between each vertex pair (thus, 14 edges in total) would also put the seven bridges of ${\mathcal {M}}_1$ into cycles. Second, observe in Fig. 6 that, for example, the addition of edges (0, 7), (1, 3), (2, 4), and (5, 6) to the graph ${\mathcal {M}}_1$ would also put all seven bridges into cycles. Thus, by adding only four new transitions (instead of 53), seven bridges of ${\mathcal {M}}_1$ can be put into cycles.

The three edges (1, 3), (2, 4), and (5, 6) correspond to three unique transitions. However, edge (0, 7) corresponds to 11 unique transitions. These 11 transitions are shown in Table 2: one endpoint of each transition is vertex #7 (with quantum numbers displayed in Fig. 6), the quantum numbers of the other endpoints are displayed in the rows of the table. Note that all quantum states shown in Table 2 correspond to vertex #0 in Fig. 6.

Under such circumstances, the transition(s) to add from the set of 11 unique transitions can be selected according to various criteria. One such criterion is selecting the transition with the highest intensity; according to this, the transition with the endpoint that is the last row of Table 2 (with a wavenumber of $1186.314\,004 \, {\hbox {cm}}^{-1}$ and an intensity magnitude of $10^{-24} \, \hbox {cm}\, {\hbox {molecule}}^{-1}$) should be selected. Another possible option is to pick the transition with the smallest uncertainty. However, in this example all the 11 unique transitions have the same uncertainty.

Using the metrics introduced in “Local and global optimality metrics”, the local optimality metric $f_{\ell }$ of this augmentation is 7, as the new edges put seven bridges into cycles. The global optimality metric $f_{\mathrm{g}}$ would depend on the theoretical spectroscopic network counterpart, which is omitted from this example for clarity.

In the example given, the min-max problem of finding the minimum number of edges to add to put the maximum number of bridges into cycles, which was found to be four, was doable by hand. In general, however, this is a difficult task for large graphs. This is where the model described in “Augmenting measured spectroscopic networks” shines. In our example, a 1.5-approximation algorithm of the Tree Augmentation Problem would highlight at most $4 \times 1.5 = 6$ new edges to add to ${\mathcal {M}}_1$ to put the seven bridges into cycles.

### A MARVEL-based application

The most recent MARVEL-based database of the $^{14} {\text {NH}}_3$ molecule contains 46 115 rovibrational transitions of experimental origin^33^. After employing a room-temperature intensity cutoff of $10^{-26} \, \hbox {cm}\, {\hbox {molecule}}^{-1}$, in effect disregarding transitions that have an intensity lower than this value means that the remaining transitions are all of considerable importance for atmospheric modeling studies, the reduced dataset contains 22214 unique transitions. This set of unique transitions is built upon a total of 4491 rovibrational energy levels.

**Table 3:** Transitions connecting the small *ortho* and *para* subgraphs with their corresponding main subgraphs and transforming bridges into cycles in the latest MARVEL database of $^{14} {\text {NH}}_3$^33^.

*w* / ${\hbox {cm}}^{-1}$	*I* / $\hbox {cm}\,{\hbox {molecule}}^{-1}$	Label of the upper state	Label of the lower state
751.2452	$1.1 \times 10^{-26}$	[0 0 0 1 0 1 17 16 a ${\hbox {E}}''$ 21]	[0 1 0 0 0 0 17 17 a ${\hbox {E}}'$ 10]
882.1105	$1.4 \times 10^{-26}$	[0 1 0 0 0 0 17 17 s ${\hbox {E}}''$ 9]	[0 0 0 0 0 0 16 14 s ${\hbox {E}}'$ 2]
937.4835	$1.3 \times 10^{-26}$	[0 1 0 0 0 0 17 17 a ${\hbox {E}}'$ 10]	[0 0 0 0 0 0 16 14 a ${\hbox {E}}''$ 2]
1343.0444	$4.0 \times 10^{-24}$	[0 0 0 1 0 1 17 16 s ${\hbox {E}}'$ 21]	[0 0 0 0 0 0 18 17 s ${\hbox {E}}''$ 1]
1343.6571	$4.0 \times 10^{-24}$	[0 0 0 1 0 1 17 16 a ${\hbox {E}}''$ 21]	[0 0 0 0 0 0 18 17 a ${\hbox {E}}'$ 1]
1355.4820	$2.1 \times 10^{-23}$	[0 0 0 1 0 1 16 16 s ${\hbox {E}}'$ 18]	[0 0 0 0 0 0 17 17 s ${\hbox {E}}''$ 1]
1355.6048	$2.1 \times 10^{-23}$	[0 0 0 1 0 1 16 16 a ${\hbox {E}}''$ 17]	[0 0 0 0 0 0 17 17 a ${\hbox {E}}'$ 1]
1515.9556	$1.1 \times 10^{-26}$	[0 2 0 0 0 0 17 17 s ${\hbox {E}}''$ 17]	[0 0 0 0 0 0 16 14 s ${\hbox {E}}'$ 2]
1542.1058	$4.3 \times 10^{-26}$	[0 0 0 1 0 1 18 15 s ${\hbox {E}}''$ 29]	[0 0 0 0 0 0 19 17 a ${\hbox {E}}'$ 2]
1551.5330	$1.3 \times 10^{-25}$	[0 0 0 1 0 1 17 15 a ${\hbox {E}}'$ 27]	[0 0 0 0 0 0 18 17 s ${\hbox {E}}''$ 1]
1565.7603	$3.1 \times 10^{-25}$	[0 0 0 1 0 1 16 15 a ${\hbox {E}}'$ 22]	[0 0 0 0 0 0 17 17 s ${\hbox {E}}''$ 1]
1569.2693	$4.5 \times 10^{-25}$	[0 0 0 1 0 1 16 15 s ${\hbox {E}}''$ 22]	[0 0 0 0 0 0 17 17 a ${\hbox {E}}'$ 1]
1573.3180	$4.1 \times 10^{-26}$	[0 2 0 0 0 0 17 14 s ${\hbox {E}}'$ 29]	[0 0 0 0 0 0 18 17 s ${\hbox {E}}''$ 1]
1584.0051	$1.4 \times 10^{-25}$	[0 2 0 0 0 0 16 14 s ${\hbox {E}}'$ 24]	[0 0 0 0 0 0 17 17 s ${\hbox {E}}''$ 1]
1697.7792	$2.6 \times 10^{-26}$	[0 0 0 1 0 1 17 16 s ${\hbox {E}}'$ 21]	[0 0 0 0 0 0 17 17 s ${\hbox {E}}''$ 1]
1906.2678	$1.6 \times 10^{-26}$	[0 0 0 1 0 1 17 15 a ${\hbox {E}}'$ 27]	[0 0 0 0 0 0 17 17 s ${\hbox {E}}''$ 1]
1910.1985	$2.2 \times 10^{-26}$	[0 0 0 1 0 1 17 15 s ${\hbox {E}}''$ 26]	[0 0 0 0 0 0 17 17 a ${\hbox {E}}'$ 1]
1913.9041	$1.1 \times 10^{-26}$	[0 0 0 1 0 1 18 15 s ${\hbox {E}}''$ 29]	[0 0 0 0 0 0 18 17 a ${\hbox {E}}'$ 1]
873.5876	$1.1 \times 10^{-26}$	[0 1 0 0 0 0 18 18 s ${\hbox {A}}_{2}'$ 5]	[0 0 0 0 0 0 17 15 s ${\hbox {A}}_{2}''$ 1]
932.0846	$1.0 \times 10^{-26}$	[0 1 0 0 0 0 18 18 a ${\hbox {A}}_{2}''$ 5]	[0 0 0 0 0 0 17 15 a ${\hbox {A}}_{2}'$ 1]
1327.8593	$2.6 \times 10^{-24}$	[0 0 0 1 0 1 18 17 a ${\hbox {A}}_{2}'$ 11]	[0 0 0 0 0 0 19 18 a ${\hbox {A}}_{2}''$ 1]
1339.2518	$1.5 \times 10^{-23}$	[0 0 0 1 0 1 17 17 s ${\hbox {A}}_{2}''$ 8]	[0 0 0 0 0 0 18 18 s ${\hbox {A}}_{2}'$ 1]
1339.3848	$1.5 \times 10^{-23}$	[0 0 0 1 0 1 17 17 a ${\hbox {A}}_{2}'$ 9]	[0 0 0 0 0 0 18 18 a ${\hbox {A}}_{2}''$ 1]
1553.3270	$1.1 \times 10^{-25}$	[0 0 0 1 0 1 18 16 s ${\hbox {A}}_{2}'$ 13]	[0 0 0 0 0 0 19 18 a ${\hbox {A}}_{2}''$ 1]
1562.7134	$2.3 \times 10^{-25}$	[0 0 0 1 0 1 17 16 a ${\hbox {A}}_{2}''$ 10]	[0 0 0 0 0 0 18 18 s ${\hbox {A}}_{2}'$ 1]
1571.0324	$2.9 \times 10^{-26}$	[0 2 0 0 0 0 18 15 s ${\hbox {A}}_{2}''$ 15]	[0 0 0 0 0 0 19 18 s ${\hbox {A}}_{2}'$ 1]
1581.4352	$1.1 \times 10^{-25}$	[0 2 0 0 0 0 17 15 s ${\hbox {A}}_{2}''$ 11]	[0 0 0 0 0 0 18 18 s ${\hbox {A}}_{2}'$ 1]
1927.1680	$1.4 \times 10^{-26}$	[0 0 0 1 0 1 18 16 s ${\hbox {A}}_{2}'$ 13]	[0 0 0 0 0 0 18 18 a ${\hbox {A}}_{2}''$ 1]

*w* and *I* are the wavenumber and the absorption intensity of the selected transition, respectively, the latter taken at room temperature. For a detailed description of the labels, see the text.

The majority of the 4491 energy levels belong to two (*ortho* and *para*) maximal 2-edge-connected subgraphs; these *ortho* and *para* ‘main subgraphs’ contain 2494 and 1292 energy levels, respectively. Within this spectroscopic network we found an additional two relatively large 2-edge-connected subgraphs which connect to their respective main subgraph by bridges. The larger subgraph of the two, containing 29 rovibrational energy levels, connects to the *ortho* main subgraph, while another subgraph, which contains 26 levels, connects to the *para* main subgraph. The algorithm described in “Augmenting measured spectroscopic networks” straightforwardly provides a set of transitions which are not currently in MARVEL, but connect the appropriate subgraph pairs. In fact, using a first-principles transition set^41^ the graph contraction algorithm selected 18 *ortho* and 10 *para* transitions, each having an intensity of at least $10^{-26} \, \hbox {cm}\,{\hbox {molecule}}^{-1}$ (at room temperature), and each connecting the two small subgraphs to their respective main subgraphs. In other words, these 18 and 10 transitions run parallel to the current bridges.

In Table 3, containing the two transition sets of size 18 and 10 suggested by our algorithm, we use the following 11 descriptors to identify rovibrational states^33^: [$v_1 \, v_2 \, v_3 \, v_4 \, L_3 \, L_4 \, J \, K \, inv \, {\varGamma }_{\mathrm{tot}} \, N_{\mathrm{block}}$], where $v_i$ ($i = 1, 2, 3, 4$) are the vibrational normal-mode quantum numbers, $L_3$ and $L_4$ are the absolute value of vibrational angular-momentum quantum numbers associated with modes 3 and 4, respectively, *J* is the total angular-momentum quantum number, $K = \left| k\right|$ is the projection of the total angular momentum on the molecule-fixed axis *z*, $inv = a /s$ is the inversion symmetry (asymmetric/symmetric or odd/even) of the vibrational motion, and ${\varGamma }_{\mathrm{tot}}$ is the full symmetry of the eigenstate. $N_{\mathrm{block}}$ is an index for the levels within the $J-{\varGamma }_{\mathrm{tot}}$ blocks of the CoYuTe^41^ energy list. By adding just one transition from each set to the current MARVEL database, the corresponding bridge becomes part of a cycle, facilitating the precise determination of the energies in the two subgraphs, as well as the detection of incorrect measurements. Clearly, the algorithm of “Augmenting measured spectroscopic networks” suggests a number of transitions with considerable intensity and in different regions of the infrared spectrum, so convenient choices can be made based on the available instrumentation and fine details of the observed spectrum.

## Conclusions

Most line-by-line spectroscopic databases undergo regular maintenance, involving expansion of the coverage offered by the database using new measurement results and improving characteristics of the existing data. During this process it is common that issues with the old and new datasets are attempted to be identified. Detecting flawed entries in line-by-line spectroscopic databases is the problem that has been addressed during this study.

Treating the transitions and the energy levels of line-by-line spectroscopic datasets as large graphs, called spectroscopic networks, opens avenues to a range of applications. For example, spectroscopic networks offer a useful framework to compare incomplete but accurate data in line-by-line spectroscopic databases to a spectroscopic network built upon complete but inaccurate first-principles data. This way not only the completeness and the validity of the entries of the spectroscopic database can be determined, but it also becomes easier to use theory to improve the actual database by, for example, suggesting new transitions to add to the database.

One of the several advantages of spectroscopic networks, utilized in this study, is that it allows the straightforward detection of flawed wavenumber entries in databases. A method^21^ that achieves this was described earlier and here it is referred to as Cycle Testing (the method was briefly recalled in “Cycle testing”). Cycle Testing is feasible only for transitions that are included in at least one cycle of the spectroscopic network. The concept of two-edge-connectivity, introduced for high-resolution spectroscopy and spectroscopic networks in this study, helps handling both the cycles and the edges that are not in cycles of the spectroscopic network. By finding the maximal two-edge-connected subgraphs of a spectroscopic network and contracting these subgraphs into single vertices, it becomes apparent which regions of the graph—which lines of the experimental database—can be covered by Cycle Testing. This graph construction also highlights if there are single transitions connecting large subgraphs. The accuracy of these transitions, called bridges, is critically important in determining accurate energies in these subgraphs, and their wavenumbers cannot be verified by Cycle Testing. This provides a motivation to try including new transitions in the database that put these bridges into cycles, making them verifiable by Cycle Testing.

A method, based on the Tree Augmentation and the Weighted Tree Augmentation problems of graph theory^24–29^, is described, which provides two-edge-connected graphs. This method allows the selection of a minimum number of new transitions to be added to an existing database, which would put the maximum number of edges, which were not in cycles before, into cycles.

To support the practical application of the results of this paper, a global and a local optimality metric are introduced. To highlight the advantages of two-edge-connectivity to spectroscopy, a synthetic experimental database and a set of extra edges is constructed from the transition list of the $^{14} {\text {NH}}_3$ molecule from the HITRAN 2016 information system^16^. First, it is shown how the contraction of the spectroscopic network works. Then, it is discussed how to select new transitions from the set of extra edges in order to put the maximum number of bridges of the spectroscopic network into cycles. Finally, an application based on the MARVEL database of $^{14} {\text {NH}}_3$ is given, whereby we suggest new transitions to add to the current experimental dataset. This would improve the accuracy of the energies of a considerable number of quantum states.
